# Supplementary material for: A fish can change its stripes: investigating the role of body colour and pattern in the bluelined goatfish
Source: PeerJ. 2024 Jan 29;12:e16645. doi: 10.7717/peerj.16645 (PMC10832622; doi:10.7717/peerj.16645)
Supplement: Supplemental Information 1 [file peerj-12-16645-s001.docx]

**Supporting Information**

A fish can change its stripes: investigating the role of colour and pattern in the bluelined goatfish

Louise Tosetto, Nathan S. Hart & Jane E. Williamson

Corresponding author: Louise Tosetto – louise.tosetto@mq.edu.au

This file includes:

Video S1 Description: Animated version of the wavefront.obj file that was generated for the 3D printed goatfish model. Model was created using Cinema4D.

Figure S1

Table S1

Table S2

Table S3

Figure S1. Spectral reflectance of A) Red and white banding from *Upeneichthys lineatus* (see Tosetto et al. 2021), B) Red and white banding of the red goatfish model C) Black and white banding of the black goatfish model. Note: the black reflectance in C is essentially zero.

Table S1. Sites with GPS coordinates of where the 3D models were deployed

| **Site** | **Zone** | **Latitude** | **Longitude** |
| --- | --- | --- | --- |
| Quarantine (Cannae Point) | Sydney | 33° 48' 53.90'' S | 151° 17' 3.96'' E |
| Rance Point | Sydney | 33° 48' 42.73'' S | 151° 17' 15.94'' E |
| Fairlight | Sydney | 33° 48' 3.60'' S | 151° 16' 20.66'' E |
| Balmoral | Sydney | 33° 49' 22.08'' S | 151° 15' 13.32'' E |
| Gordons Bay | Sydney | 33° 54' 56.16'' S | 151° 15' 44.64'' E |
| Bare Island | Botany Bay | 33° 59' 31.92'' S | 151° 13' 48.36'' E |
| Browns Rock | Botany Bay | 33° 59' 40.92'' S | 151° 14' 12.84'' E |
| Callala Bay | Jervis Bay | 35° 0' 36.36'' S | 150° 43' 29.64'' E |
| Hyams | Jervis Bay | 35° 6' 42.48'' S | 150° 42' 38.88'' E |
| Orion | Jervis Bay | 35° 3' 31.32'' S | 150° 41' 29.4'' E |
| Dent Rock | Jervis Bay | 35° 4' 0.12'' S | 150° 43' 0.12'' E |
| Blemheim | Jervis Bay | 35° 4' 46.2'' S | 150° 42' 4.68'' E |
| Moona | Jervis Bay | 35° 2' 44.88'' S | 150° 41' 3.48'' E |
| Pretty Beach | Kioloa | 35° 34' 24.96'' S | 150° 21' 59.04'' E |
| Yallumgo Cove | Eden | 37° 4' 9.84'' S | 149° 54' 42.48'' E |

Table S2. Pairwise comparisons for the main effects (3D model colour and fish behaviour) for Upeneichthys lineatus

| Main Effect | *t* | *P* |
| --- | --- | --- |
| **Behaviour** |  |  |
| **Foraging v Attracted** | **6.266** | **<0.001** |
| Foraging v Passing | 3.859 | 0.015 |
| **Passing v Attracted** | **3.859** | **<0.001** |
|  |  |  |
| **Colour** |  |  |
| Black v White | 2.395 | 0.045 |
| **Red v White** | **2.799** | **0.015** |
| Red v Black | 0.255 | 0.965 |

Table S3. Pairwise comparisons for the main effects (3D model colour and behaviours) for each of the nine key heterospecifics

|  | *Chrysophrys auraus* | | *Scobinichthys granulatus* | | *Gerres subfasciatus* | | *Ophthalmolepsis lineolata* | | *Acanthopagrus australis* | | *Atypicthys strigatus* | | *Parupeneus spilurus* | | *Eupetrichthys angustipes* | | *Pseudocaranx georgianus* | |  |
| --- | --- | --- | --- | --- | --- | --- | --- | --- | --- | --- | --- | --- | --- | --- | --- | --- | --- | --- | --- |
|  |  |  |  |  |  |  |  |  |  |  |  |  |  |  |  |  |  |  |  |
|  | z | *P* | *z* | *P* | z | *P* | z | *P* | z | *P* | z | *P* | z | *P* | z | *P* | z | *P* |  |
| **Colour x Activity** | - | 0.729 | - | 0.782 | - | 0.999 | - | 0.664 | - | 0.577 | - | 0.964 | - | 0.573 | - | 0.856 |  | 0.949 |  |
| **Colour** |  |  |  |  |  |  |  |  |  |  |  |  |  |  |  |  |  |  |  |
| Black v White | -0.985 | 0.586 | -1.981 | 0.117 | -1.044 | 0.549 | 0.545 | 0.849 | 0.879 | 0.653 | -0.774 | 0.719 | 0.794 | 0.707 | -1.208 | 0.449 | 0.025 | 0.100 |  |
| Red v White | -0.673 | 0.779 | -0.296 | 0.953 | -0.397 | 0.917 | 0.896 | 0.643 | 0.152 | 0.987 | -1.114 | 0.505 | 0.694 | 0.767 | 1.065 | 0.537 | 0.130 | 0.991 |  |
| Red v Black | 0.361 | 0.931 | 1.752 | 0.186 | 0.689 | 0.770 | 0.310 | 0.949 | -0.758 | 0.729 | -0.287 | 0.956 | -0.143 | 0.989 | 2.280 | 0.060 | 0.100 | 0.994 |  |
|  |  |  |  |  |  |  |  |  |  |  |  |  |  |  |  |  |  |  |  |
| **Behaviour** |  |  |  |  |  |  |  |  |  |  |  |  |  |  |  |  |  |  |  |
| Feeding v Attracted | -1.801 | 0.169 | -0.292 | 0.954 | 1.502 | 0.290 | **-3.133** | **0.005** | 1.086 | 0.523 | **-3.345** | **0.002** | -1.508 | 0.287 | -2.079 | 0.096 | -0.724 | 0.750 |  |
| Passing v Attracted | -1.142 | 0.488 | 0.450 | 0.894 | 1.042 | 0.550 | -0.803 | 0.701 | 1.509 | 0.286 | -1.313 | 0.387 | 0.637 | 0.800 | 2.343 | 0.051 | **3.643** | **<0.001** |  |
| Passing v Feeding | 0.764 | 0.725 | 0.750 | 0.734 | -0.549 | 0.847 | **2.494** | **0.034** | 0.348 | 0.936 | 2.215 | 0.070 | 2.211 | 0.070 | **4.488** | **<0.001** | **4.351** | **<0.001** |  |

Table S4. Pairwise comparisons of the time spent around the different 3D model coloured fish by the nine key heterospecifics

|  | | *Chrysophrys auraus* | | | *Scobinichthys granulatus* | | | *Gerres subfasciatus* | | | *Ophthalmolepsis lineolata* | | | *Acanthopagrus australis* | | | *Atypicthys strigatus* | | | *Parupeneus spilurus* | | | *Eupetrichthys angustipes* | | | *Pseudocaranx georgianus* | | | |
| --- | --- | --- | --- | --- | --- | --- | --- | --- | --- | --- | --- | --- | --- | --- | --- | --- | --- | --- | --- | --- | --- | --- | --- | --- | --- | --- | --- | --- | --- |
| **Colour** |  | |  |  | |  |  | |  |  | |  |  | |  |  | |  |  | |  |  | |  |  | |  |  |  |
| Red v Black | **3.083** | | **0.006** | **2.630** | | **0.038** | -1.694 | | 0.210 | 0.777 | | 0.717 | 1.915 | | 0.196 | -0.197 | | 0.979 | -1.137 | | 0.502 | -1.114 | | 0.503 | -1.042 | | 0.555 |  |  |
| White v Black | 0.880 | | 0.652 | 0.746 | | 0.731 | **-2.567** | | **0.029** | **2.432** | | **0.041** | **3.705** | | **0.014** | 1.925 | | 0.146 | -0.945 | | 0.619 | -1.320 | | 0.381 | -1.758 | | 0.196 |  |  |
| White v Red | **-2.603** | | **0.025** | **-3.071** | | **0.014** | -1.154 | | 0.482 | 1.716 | | 0.201 | 2.344 | | 0.102 | 2.096 | | 0.101 | 0.087 | | 0.996 | -0.209 | | 0.976 | -1.115 | | 0.511 |  |  |
